# Different sodium concentrations of noncancerous and cancerous prostate tissue seen on MRI using an external coil

**DOI:** 10.1093/radadv/umae023

**Published:** 2024-09-30

**Authors:** Josephine L Tan, Vibhuti Kalia, Stephen E Pautler, Glenn Bauman, Lena V Gast, Max Müller, Armin M Nagel, Jonathan D Thiessen, Timothy J Scholl, Alireza Akbari

**Affiliations:** Medical Biophysics, Western University, London, N6A 3K7, Canada; Medical Imaging, Western University, London, N6A 3K7, Canada; Medical Imaging, St Joseph's Health Care, London, N6A 4V2, Canada; Surgery, Western University, London, N6A 3K7, Canada; Oncology, Western University, London, N6A 3K7, Canada; Medical Biophysics, Western University, London, N6A 3K7, Canada; Oncology, Western University, London, N6A 3K7, Canada; Institute of Radiology, University Hospital Erlangen, Friedrich‐Alexander‐Universität (FAU) Erlangen‐Nürnberg, Erlangen, 91054, Germany; Institute of Radiology, University Hospital Erlangen, Friedrich‐Alexander‐Universität (FAU) Erlangen‐Nürnberg, Erlangen, 91054, Germany; Institute of Radiology, University Hospital Erlangen, Friedrich‐Alexander‐Universität (FAU) Erlangen‐Nürnberg, Erlangen, 91054, Germany; Medical Physics in Radiology, German Cancer Research Center (DKFZ), Heidelberg, 69120, Germany; Medical Biophysics, Western University, London, N6A 3K7, Canada; Medical Imaging, Western University, London, N6A 3K7, Canada; Lawson Imaging, Lawson Health Research Institute, London, N6A 5W9, Canada; Medical Biophysics, Western University, London, N6A 3K7, Canada; Robarts Research Institute, London, N6A 3K7, Canada; Ontario Institute for Cancer Research, Toronto, M5G 0A3, Canada; Medical Biophysics, Western University, London, N6A 3K7, Canada; Lawson Imaging, Lawson Health Research Institute, London, N6A 5W9, Canada; Robarts Research Institute, London, N6A 3K7, Canada

**Keywords:** tissue sodium concentration, apparent diffusion coefficient, human prostate cancer, 3 Tesla, sodium (23Na) MRI

## Abstract

**Background:**

Sodium (^23^Na) MRI of prostate cancer (PCa) is a novel but underdocumented technique conventionally acquired using an endorectal coil. These endorectal coils are associated with challenges (e.g., a nonuniform sensitivity profile, limited prostate coverage, patient discomfort) that could be mitigated with an external ^23^Na MRI coil.

**Purpose:**

To quantify tissue sodium concentration (TSC) differences within the prostate of participants with PCa and healthy volunteers using an external ^23^Na MRI radiofrequency coil at 3 T.

**Materials and Methods:**

A prospective study was conducted from January 2022 to June 2024 in healthy volunteers and participants with biopsy-proven PCa. Prostate ^23^Na MRI was acquired on a 3-T PET/MRI scanner using a custom-built 2-loop (diameter, 18 cm) butterfly surface coil tuned for the ^23^Na frequency (32.6 MHz). The percent difference in TSC (ΔTSC) between prostate cancer lesions and surrounding noncancerous prostate tissue of the peripheral zone (PZ) and transition zone (TZ) was evaluated using a 1-sample *t*-test. TSC was compared to apparent diffusion coefficient (ADC) measurements as a clinical reference.

**Results:**

Six healthy volunteers (mean age, 54.5 years ± 12.7) and 20 participants with PCa (mean age, 70.7 years ± 8.3) were evaluated. A total of 31 lesions were detected (21 PZ, 10 TZ) across PCa participants. Compared to noncancerous prostate tissue, prostate cancer lesions had significantly lower TSC (ΔTSC, –14.1% ± 18.2, *P* = .0002) and ADC (ΔADC, –26.6% ± 18.7, *P* < .0001).

**Conclusion:**

We used an external ^23^Na MRI coil for whole-gland comparison of TSC in PCa and noncancerous prostate tissue at 3 T. PCa lesions presented with lower TSC compared to surrounding noncancerous PZ and TZ tissue. These findings demonstrate the feasibility of an external ^23^Na MRI coil to quantify TSC in the prostate and offer a promising, noninvasive approach to PCa diagnosis and management.

**Abbreviations**

ADC = apparent diffusion coefficient, FOV = field of view, mpMRI = multiparametric MRI, PCa = prostate cancer, PIRADS = Prostate Imaging Reporting and Data System, PZ = peripheral zone, ROI, region of interest, SNR = signal-to-noise ratio, TE = echo time, TR, repetition time, TSC = tissue sodium concentration, TZ = transition zone

**Summary**

MRI was used to compare tissue sodium concentration in prostate cancer lesions to noncancerous tissue with a novel external sodium-23 (^23^Na) radiofrequency coil at 3 Tesla.

**Key Results**

- The external coil detected sodium signal throughout the whole prostate (SNR ∼ 30).
- Sodium concentration was significantly different between cancers and noncancerous tissue in the transition zone (64.0 mM ± 15.1 vs. 80.9 mM ± 16.3, *P* = .0217), but not in the peripheral zone (70.3 mM ± 16.0 vs. 78.2 mM ± 14.1, *P* = .235).
- Based on percent difference, cancers had significantly lower sodium concentrations (–14.1% ± 18.2, *P* = .0002) compared to noncancerous tissue.

## Introduction

Prostate biopsy is the gold standard for prostate cancer (PCa) diagnosis and allows assignment of Gleason grade, a key factor for predicting clinical behavior and guiding treatment. However, a typical biopsy samples less than 1% of the prostate gland, which poses a high risk of missing clinically significant tumour foci or underestimating Gleason grade.^1^ Clinical imaging techniques such as proton (^1^H)-based multiparametric MRI (mpMRI) have improved the identification and targeting of potentially clinically significant PCa^2^ with established qualitative scoring systems (e.g., Prostate Imaging Reporting and Data System [PIRADS]^3^) but their diagnostic accuracy and ability to characterize cancer grade remains imperfect. Therefore, there is a critical need for a noninvasive diagnostic tool that can supplement the inherent limitations of conventional mpMRI.

Several prostate tumor types including PCa exhibit altered tissue sodium concentration (TSC), which can be detected on sodium (^23^Na) MRI^4–6^ as apparent TSC. The term apparent TSC accounts for biases in the measured TSC resulting from relaxation and pulse sequence characteristics.^7^^,^^8^ Using ^23^Na MRI, TSC has been quantified in noncancerous prostate tissue and PCa^9–11^ and has been significantly correlated to histological Gleason grade.^9^ In contrast to mpMRI, the routine use of ^23^Na MRI in the clinic is largely limited because of its low signal-to-noise ratio (SNR). Low ^23^Na signal arises from its low biological abundance (∼2000× lower than ^1^H), low gyromagnetic ratio (∼4× lower than ^1^H), and quadrupolar nuclear moment (spin 3/2), the latter of which leads to a rapid biexponential signal decay (T_2, long_ = 14-30 ms, T_2, short_ = 0.5-5 ms, T_1_ = 10-40 ms).^4–6^

To facilitate a higher SNR in PCa imaging at clinical field strengths (1.5 T and 3 T),^23^Na MRI is typically performed using an endorectal coil.^9–12^ However, the sensitivity profiles of these surface coils are inherently nonuniform with a limited field of view, resulting in substantial signal dropoff away from the coil center. Other complications associated with endorectal coils include patient discomfort, gland deformation, improper coil positioning, and ejection of the coil from the rectum. These factors restrain the workflow of this technique and confound image interpretability, warranting the development and evaluation of an external ^23^Na coil to simplify the application of ^23^Na MRI in human PCa studies.

To address these challenges, we have developed a completely external “butterfly” coil for ^23^Na MRI of PCa at 3 T. To our knowledge, only 2 other studies have employed an external coil for ^23^Na MRI of the prostate, in which TSC was quantified in a healthy, young population (age = 29 years ± 2) and compared to apparent diffusion coefficient (ADC) measurements,^13^ and in men with clinically suspected PCa using the femoral blood vessels as internal references.^14^ In our study, we extend these prior works by quantifying TSC in noncancerous tissue and PCa in an older population with biopsy-proven PCa using ^23^Na MRI. We hypothesized that quantification of TSC differences throughout the whole prostate of participants with PCa and healthy volunteers is feasible at 3 T using our external ^23^Na MRI radiofrequency coil.

## Materials and methods

### Study design and participants

Six healthy male volunteers and 20 participants with biopsy-proven PCa were recruited from 2 exploratory, ongoing prospective single-center studies (Lawson Health Research Institute, London, Canada) approved by the institutional review board (NCT05269550, NCT04053842). These studies were conducted from January 2022 to June 2024. Written informed consent was obtained from all participants.

Key inclusion criteria were chosen to target an intermediate-high risk population defined by a Gleason Grade Group of at least 3. Key exclusion criteria included prior PCa treatment, contraindication to MRI, acute kidney injury, chronic kidney disease stage 4 or 5, and poor baseline urinary function. Study participants underwent prostate-specific antigen blood testing and a single imaging session consisting of ^23^Na MRI and mpMRI.

### MR imaging

**Figure 1.**
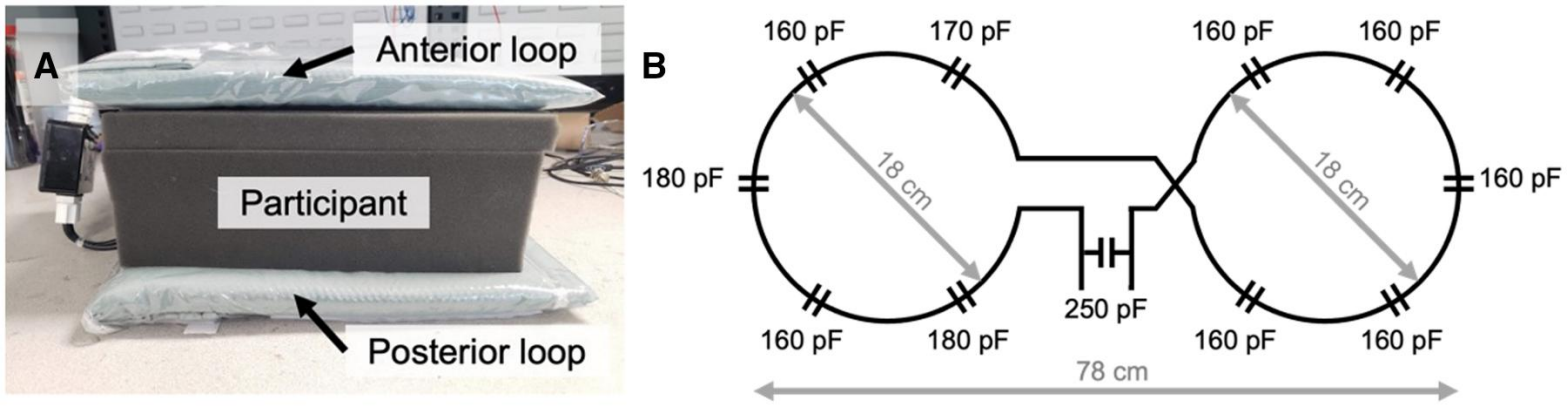
(A) External, flexible sodium (^23^Na) transmit/receive butterfly coil (tuning = 32.6 MHz) consisting of an anterior and posterior loop around the participant. (B) Circuit diagram of the butterfly coil.

MRI was performed using a 3 Tesla PET/MRI scanner (Siemens Biograph mMR). The radiofrequency system included an external, flexible transmit/receive butterfly coil consisting of 2 loops (diameters = 18 cm, tuning = 32.6 MHz) built in-house for ^23^Na imaging (Figure 1).^15^ A commercial receive-only anterior and spine array were used for ^1^H imaging (Siemens Healthcare Limited, Erlangen, Germany). The ^23^Na coil housing included 3 vials of sodium chloride solution (50, 75, 100 mM) to serve as external reference standards for quantifying TSC.

Sodium imaging was performed using a 3-dimensional density-adapted radial projection sequence^15^ (repetition time [TR] = 50 ms; echo time [TE]  = 0.8 ms; nominal flip angle = 60°; nominal isotropic resolution = 5 mm; excitation pulse width = 1 ms; field of view [FOV] = 360 × 360 × 100 mm^3^; number of projections = 11,310, samples per projection = 2,500; acquisition window = 25 ms; repetitions = 2, total scan time = 19 min), which has been previously applied in clinical research such as in multiple sclerosis^16^ and kidney disease.^17^ Quantitative assessment of the pulse sequence resolution is described in the supplemental material (Appendix S1, Figure S1, and Table S1). Three-dimensional T_2_W images were acquired as an anatomical reference (TR = 1700 ms, TE = 104 ms, FOV = 320 × 320 × 72 mm^3^, resolution = 0.7 × 0.7 × 1.0 mm^3^). Diffusion-weighted imaging was performed to generate ADC maps (TR = 5100 ms, TE = 93 ms, FOV = 220 × 200 mm^2^, resolution = 2.5 × 2.5 mm^2^, slice thickness = 3 mm, number of slices = 24, b-values = 0, 200, 500, 1000, 1500, 2000 s/mm^2^). The full MRI protocol of primary sequences is included in the supplemental material (Table S2).

^23^Na MRI was first performed on healthy male volunteers and participants with biopsy-proven PCa. Following ^23^Na MRI, the butterfly coil was removed and the anterior and spine arrays were placed for ^1^H-based T_2_W and diffusion-weighted imaging MRI. ^23^Na MRI was acquired in a straight axial plane, whereas mpMRI was acquired in an oblique axial plane (perpendicular to the prostatic urethra).

### Sodium image postprocessing

^23^Na MR images were reconstructed offline using the Michigan Image Reconstruction Toolbox (Fessler JA, www.web.eecs.umich.edu/∼fessler/code) in MATLAB. Sensitivity correction and generation of TSC maps for each participant were performed as described in the supplemental material (Appendix S2 and Figure S2).^12^^,^^18^ Validation of TSC measurements were performed in a calibration phantom as described in the supplemental material (Appendix S3 and Figure S3).^15^

### TSC measurements

A radiologist with 6 years of experience in pelvic imaging (V.K.) assessed the mpMRI datasets to (1) delineate 3 regions of interest (ROIs): the noncancerous peripheral zone (PZ), transition zone (TZ), and PCa lesions; and 2) assign the lesions a PIRADS score.^3^ ROIs were identified and delineated on all slices containing the prostate of the mpMRI image that best showed anatomical differentiation. In almost all cases, the high-resolution T_2_W image was chosen. TZ delineations included the central zone of the prostate as the 2 regions cannot be distinguished on mpMRI.^19^ Radiologist readings were performed blind to ^23^Na MR images and biopsy Gleason grades.^12^^,^^18^ The mean TSC and ADC were quantified in the PZ, TZ, and lesions. The percent differences in TSC (ΔTSC) and ADC (ΔADC) between lesions and noncancerous tissue were also calculated in PCa participants^9^:

$$\Delta \mathrm{TSC}=\frac{\mathrm{TS}C_{\mathrm{lesion}}-\mathrm{TS}C_{\mathrm{noncancerous}}}{(\mathrm{TS}C_{\mathrm{lesion}}+\mathrm{TS}C_{\mathrm{noncancerous}})/2}*100\%$$

$$\Delta \mathrm{ADC}=\frac{\mathrm{AD}C_{\mathrm{lesion}}-\mathrm{AD}C_{\mathrm{noncancerous}}}{({\mathrm{ADC}}_{\mathrm{lesion}}+{\mathrm{ADC}}_{\mathrm{noncancerous}})/2}*100\%$$

Compared to absolute measurements of TSC, percent differences may better account for TSC fluctuations within and between individuals depending on age, time-of-day, and diet.^20–22^

### Statistical analysis

A 1-way analysis of variance and Šídák multiple comparison test were used to compare mean absolute TSC and ADC values in (1) noncancerous PZ between healthy volunteers and PCa participants, (2) noncancerous TZ between healthy volunteers and PCa participants, (3) between PCa lesions and noncancerous tissue in the PZ of PCa participants, and (4) between PCa lesions and noncancerous tissue in the TZ of PCa participants. For each comparison, a *P* value < .05 was deemed statistically significant. ΔTSC and ΔADC were evaluated using a 1-sample *t*-test against a hypothetical mean value of 0% (indicating no difference between noncancerous tissue and tumor lesion), where a *P* value < .05 was deemed statistically significant. All statistical analysis was performed using GraphPad Prism version 10.2.3, GraphPad Software, Boston, MA, USA, www.graphpad.com.

## Results

**Figure 2.**
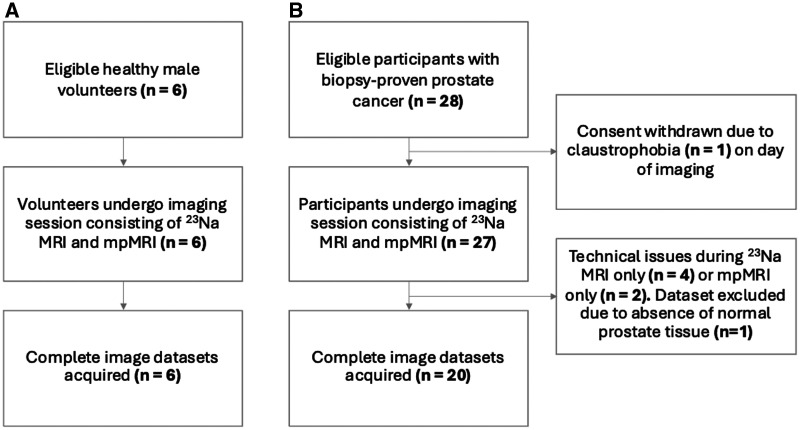
Flowchart shows (A) healthy volunteers and (B) participants with prostate cancer who underwent a single imaging session consisting of sodium (^23^Na) MRI and multiparametric MRI (mpMRI).

As of our study’s submission, 6 healthy male volunteers and 28 PCa participants were recruited for this study (Figure 2). Complete image datasets were collected for all 6 volunteers and for 20 PCa participants (Table 1). The mean age was 54.5 years ± 12.7 (range = 45–76 years) in volunteers and 70.7 years ± 8.3 (range = 54–85 years) in PCa participants. PCa participants had a mean PSA level of 17.6 ng/mL ± 14.5 and Gleason grades of 3 + 4 (n = 7), 4 + 3 (n = 9), and 4 + 4 (n = 4) determined at biopsy before imaging. Across all PCa participants, a total of 31 tumor lesions were detected (21 PZ, 10 TZ) by mpMRI with PIRADS score 3 (n = 3), 4 (n = 12), and 5 (n = 16). PCa lesions had a mean volume of 2.5 cm^3^ ± 3.3.

**Table 1.** Participant characteristics (numbers represent mean ± standard deviation unless otherwise indicated).

Parameter	Participants with PCa	Healthy volunteers
Number of participants	20	6
Age (years)	70.7 ± 8.3	54.5 ± 12.7
Body mass index (kg/m^2^)	28.7 ± 6.5	24.8 ± 3.0
Pelvis thickness (cm)	22.3 ± 3.3	20.3 ± 1.4
PSA (ng/mL)	17.6 ± 14.5	–
Gleason grade (from biopsy)		
3 + 3	0	–
3 + 4	9	–
4 + 3	7	–
4 + 4	4	–
Number of lesions	31	–
Lesion location		
PZ	21	–
TZ	10	–
Lesion volume (cm^3^)	2.5 ± 3.3	–
Lesion PIRADS score		
3	3	–
4	12	–
5	16	–

Abbreviations: PCa = prostate cancer, PSA = prostate-specific antigen, PZ = peripheral zone, TZ = transition zone, PIRADS = Prostate Imaging Reporting and Data System.

**Figure 3.**
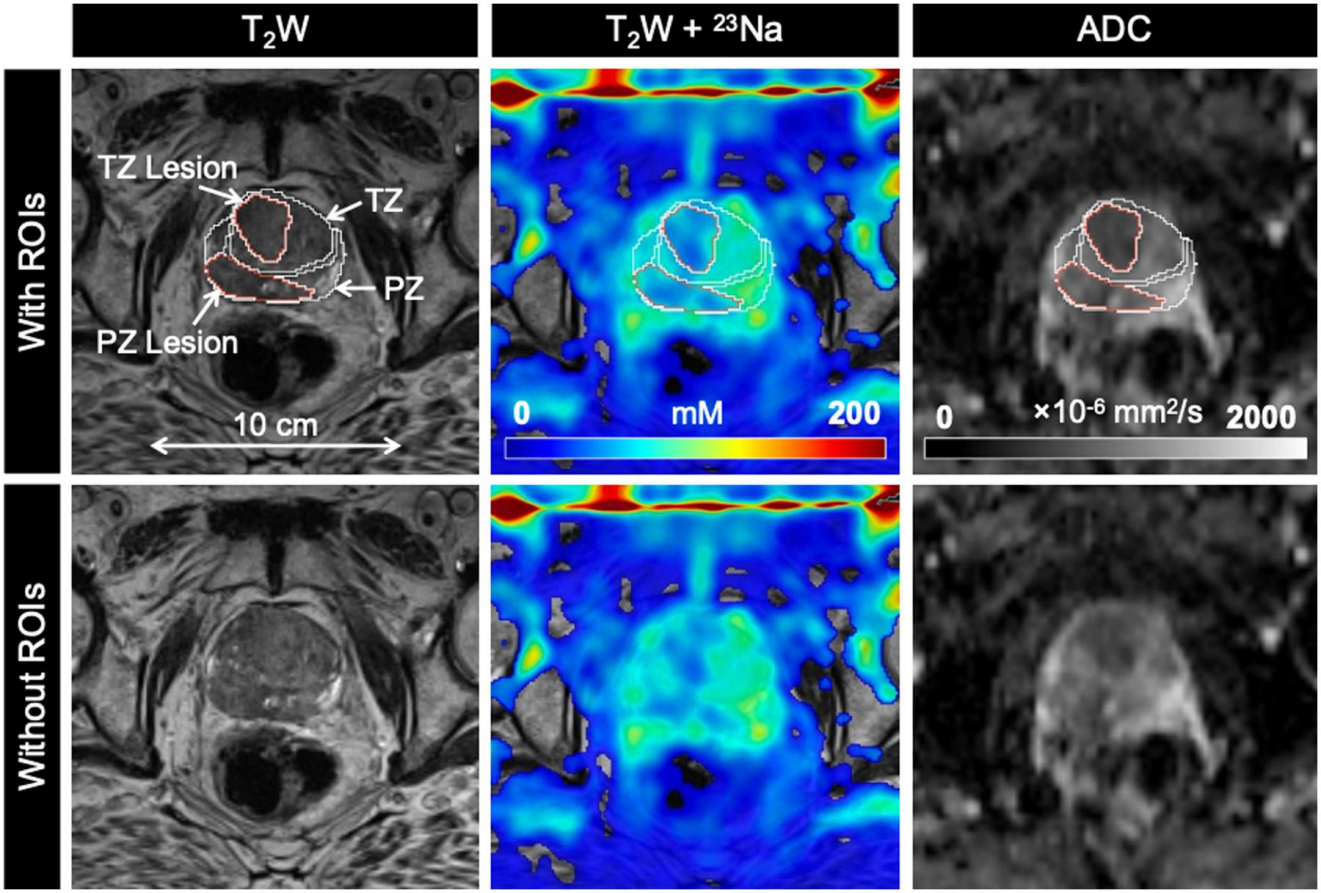
Axial T_2_-weighted (T_2_W), sodium (^23^Na), and apparent diffusion coefficient (ADC) images in a 77-year-old man with biopsy-proven prostate cancer (Gleason score 4 + 4). PZ and TZ lesions (both PIRADS score 5) show lower tissue sodium concentration (TSC) and ADC relative to surrounding noncancerous tissue. Abbreviations: PIRADS = Prostate Imaging Reporting and Data System, PZ = peripheral zone, ROIs = regions of interest, TZ = transition zone.

**Figure 4.**
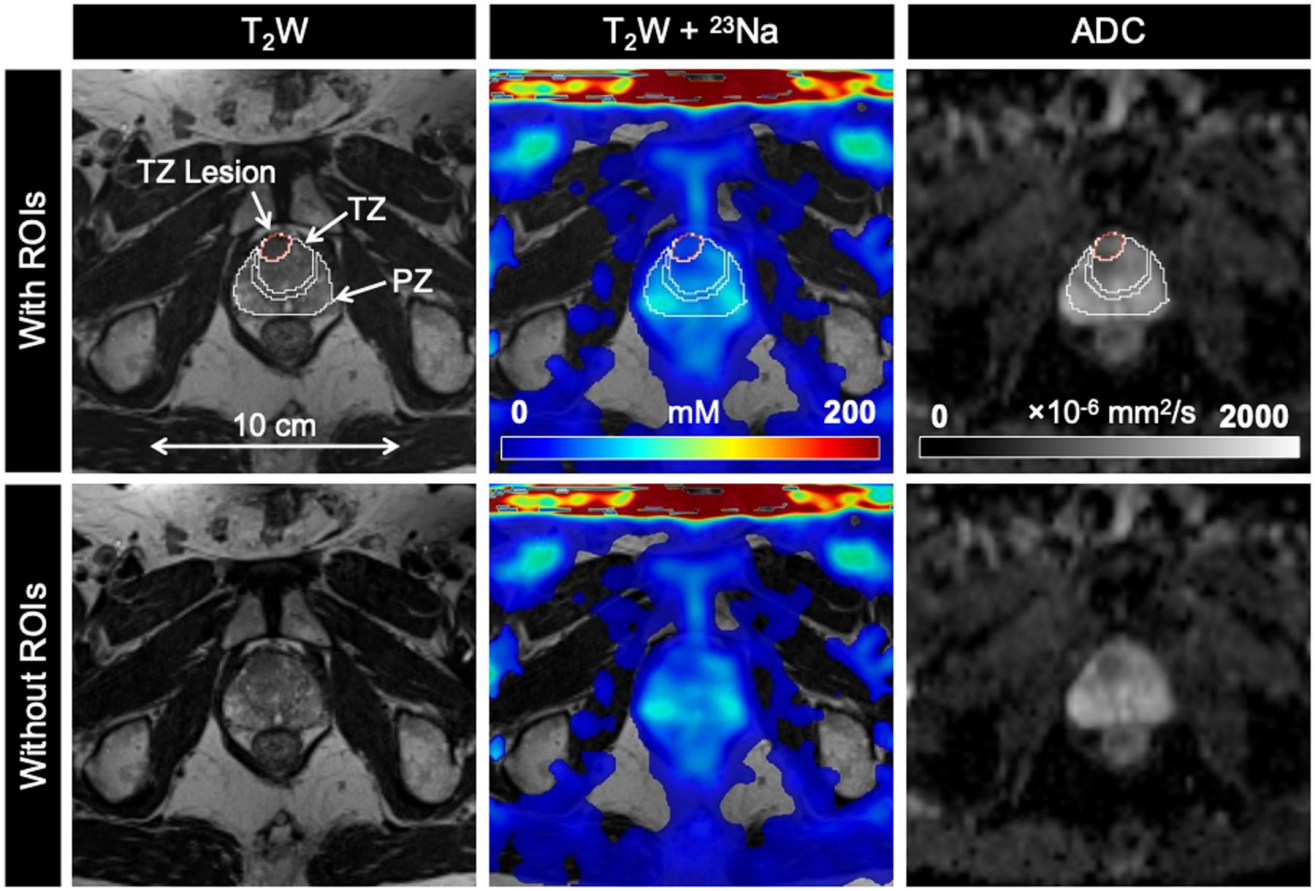
Axial T_2_-weighted (T_2_W), sodium (^23^Na), and apparent diffusion coefficient (ADC) images in a 65-year-old man with biopsy-proven prostate cancer (Gleason score 3 + 4). TZ lesion (PIRADS score 5) shows lower tissue sodium concentration (TSC) and ADC relative to surrounding noncancerous tissue. Abbreviations: PIRADS = Prostate Imaging Reporting and Data System, PZ = peripheral zone, ROIs = regions of interest, TZ = transition zone.

**Figure 5.**
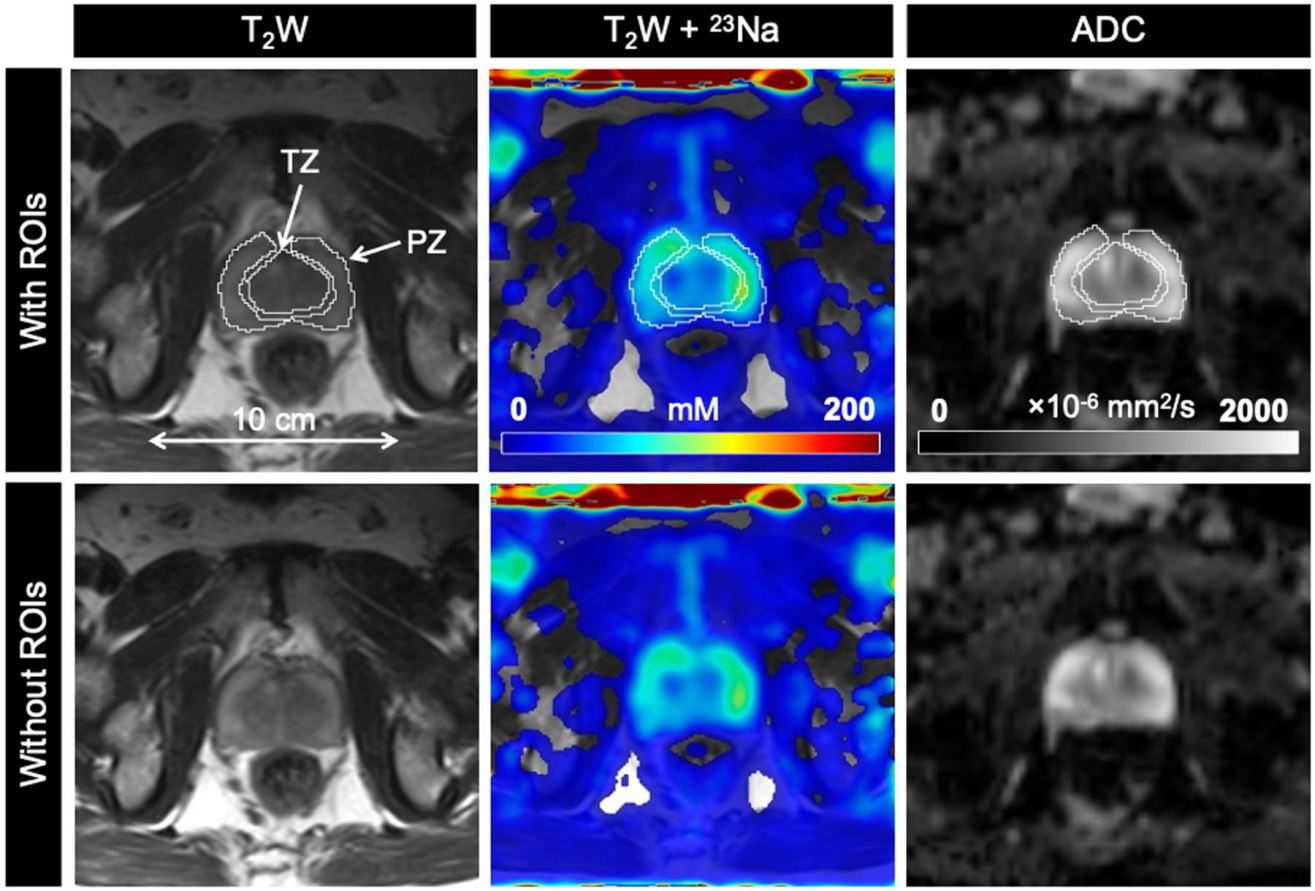
Axial T_2_-weighted (T_2_W), sodium (^23^Na), and apparent diffusion coefficient (ADC) images in a 63-year-old healthy man. Abbreviations: PZ = peripheral zone, ROIs = regions of interest, TZ = transition zone.

**Figure 6.**
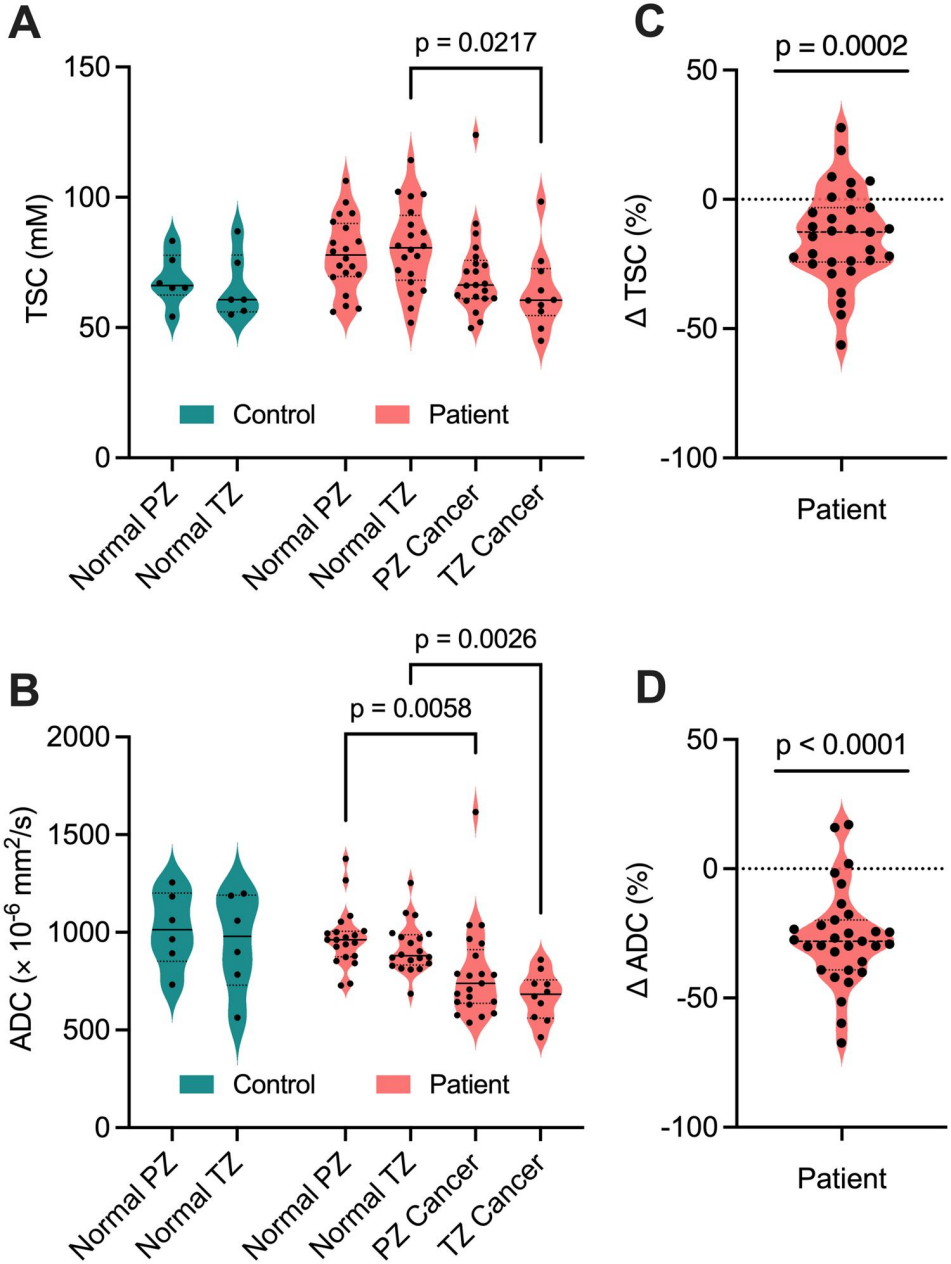
(A) Tissue sodium concentration (TSC) and (B) apparent diffusion coefficient (ADC) measurements in noncancerous (normal) tissue and cancerous lesions in the peripheral zone (PZ) and transition zone (TZ) of 6 healthy controls and 20 participants with prostate cancer. Across the participants, a total of 31 tumor lesions were detected (21 PZ, 10 TZ) by multiparametric MRI. Percent differences in (C) TSC (ΔTSC) and (D) ADC (ΔADC) in prostate cancer participants were significantly less than 0% (*P* < .05), indicating a lower TSC and ADC in PZ and TZ lesions relative to surrounding noncancerous tissue on an individual basis.

mpMRI and ^23^Na MRI setup and image acquisition were each approximately 30 minutes in duration, for a total average imaging time of 1 hour. Individual PCa participant and volunteer imaging data are listed in the supplemental material (Tables S3 and S4). Sodium signal was detected throughout the whole prostate and was also observed in the bladder, iliac arteries, spermatic cord, and rectum of all healthy volunteers and PCa participants. Sodium SNR in the prostate was consistently above 30. TSC was measured in the noncancerous PZ, noncancerous TZ, and tumor lesions (Figures 3-5, Table 2). Based on absolute measurements of TSC, there were no significant differences in TSC of noncancerous tissue between volunteers (PZ: 68.4 mM ± 10.0, TZ: 65.7 mM ± 12.5) and PCa participants (PZ: 78.2 mM ± 14.1, TZ: 80.9 mM ± 16.3) (Figure 6). In PCa participants alone, there was no significant difference in TSC between noncancerous PZ and PZ lesions (PZ lesions: 70.3 mM ± 16.0) but a significant difference in TSC between noncancerous TZ and TZ lesions (TZ lesions: 64.0 mM ± 15.1, *P* = .0217). Based on the percent difference between noncancerous prostate tissue and PCa lesions, PCa lesions had significantly lower TSC (ΔTSC, –14.1% ± 18.2, *P* = .0002).

**Table 2.** Mean tissue sodium concentration (TSC) and apparent diffusion coefficient (ADC) values for noncancerous tissues of the peripheral zone (PZ), transition zone (TZ), and tumors in the PZ and TZ.

Parameter	Participants with PCa	Healthy volunteers	*P* value
TSC (mM)			
PZ	78.2 ± 14.1	68.4 ± 10.0	0.0512
TZ	80.9 ± 16.3	65.7 ± 12.5	0.0512
PZ lesion	28.7 ± 6.5	–	0.235
TZ lesion	22.3 ± 3.3	–	0.0217*
Δ**TSC**	−14.0 ± 18.2	–	0.0002*
ADC (×10^−6^ mm^2^/s)			
PZ	970 ± 152	1015 ± 193	0.949
TZ	918 ± 127	949 ± 249	0.949
PZ lesion	791 ± 242	–	0.0058*
TZ lesion	673 ± 123	–	0.0026*
ΔADC	−26.6 ± 18.7	–	<0.0001*

Percent differences in TSC (ΔTSC) and ADC (ΔADC) between lesions and noncancerous tissue are listed for participants with prostate cancer (PCa). A 1-way analysis of variance and Tukey test were used to compare noncancerous PZ and TZ between PCa participants and volunteers and noncancerous PZ and TZ between PZ lesions and TZ lesions in PCa participants. A 1-sample *t*-test was used to compare ΔTSC and ΔADC against a hypothetical mean value of 0%.

* A statistically significant difference (*P* < .05).

ADC was also measured in the noncancerous PZ, noncancerous TZ, and tumor lesions (Figures 3-5, Table 2). Based on absolute measurements of ADC, there were no significant differences in ADC of noncancerous tissue between volunteers (PZ: 1020 × 10^−6^ mm^2^/s ± 193, TZ: 949 × 10^−6^ mm^2^/s ± 249) and PCa participants (PZ: 969 × 10^−6^ mm^2^/s ± 152, TZ: 918 × 10^−6^ mm^2^/s ± 127) (Figure 6). In PCa participants alone, there was a significant difference in ADC between noncancerous PZ and PZ lesions (PZ lesions: 791 × 10^−6^ mm^2^/s ± 242, *P* = .0058), and between noncancerous TZ and TZ lesions (TZ lesions: 673 × 10^−6^ mm^2^/s ± 123, *P* = .0026). Based on the percent difference between noncancerous prostate tissue and PCa lesions, PCa lesions had significantly lower ADC (ΔADC, –26.6% ± 18.7, *P* < .0001).

## Discussion

In this work, we evaluated the feasibility of an external ^23^Na butterfly coil built in-house to quantify TSC in participants with PCa and in healthy volunteers. ^23^Na image setup and acquisition were 30 minutes, for a total scan time of 1 hour including conventional mpMRI. In both groups, mean TSC and ADC values reflected those reported in previous studies.^10–13^^,^^23^^,^^24^ Sodium concentration was significantly different between cancers and noncancerous tissue in the TZ but not in the PZ. Based on percent difference, cancers had significantly lower sodium concentrations (–14.1% ± 18.2, *P* = .0002) compared to noncancerous tissue.

Differences in TSC and ADC can be attributed to structural differences in these zones that influence intra- and extracellular volume fraction which, in turn, affect total TSC and water diffusivity.^4^^,^^25^^,^^26^ For example, PZ is composed of relatively looser stroma and a larger extracellular space.^25^ Relative to TZ, this tissue composition leads to an increase in TSC because of the larger contribution of extra- compared to intracellular sodium concentration (135-150 mM vs. 10-15 mM)^4^ and an increase in ADC because of the freer diffusion of water molecules. Meanwhile, PZ and TZ tumor lesions had significantly lower TSC and ADC relative to surrounding noncancerous tissue in PCa participants based on percent differences. Compared to absolute measurements of TSC and ADC, these relative measurements account for potential fluctuations in sodium, and, by extension, in water content and diffusion on an individual basis. Lower tumor TSC and ADC are in support of the high cellularity characteristic of many tumor types including PCa. Specifically, the denser cellular environment would decrease extracellular volume fraction (decreasing TSC) and restrict water diffusion (decreasing ADC) as described previously. The molecular information provided by ^23^Na MRI may supplement the inherent limitations of conventional ^1^H-based mpMRI, especially in regions beyond the PZ of the prostate because of the low intrinsic contrast between tumor and noncancerous tissue, and in the presence of conditions such as benign prostatic hyperplasia, hemorrhage, and prostatitis that may mimic the image characteristics of PCa.

Contrary to our observation of low TSC in PCa lesions, other ^23^Na MRI studies have reported mean increased TSC in PCa compared to noncancerous tissue^10^^,^^11^ as well as a significant correlation between TSC and Gleason grade.^9^ More recently, Adlung et al. showed lower TSC in PCa compared to contralateral regions.^14^ These conflicting findings suggest that changes in tumor TSC cannot solely be attributed to differences in cellular density, and other factors such as cell membrane integrity and metabolic activity should be considered.^10^ It must also be noted that there is intra- and intertumoral heterogeneity in TSC, with some prostatic tumors exhibiting lower TSC compared to noncancerous PZ and TZ.^9^^,^^10^ Finally, it is still unclear whether our results can be directly compared to current ^23^Na MRI studies of PCa, all of which have employed endorectal coils and vary in methods for sensitivity correction and TSC quantification. A study comparing the quality and performance of ^23^Na MRI with an endorectal coil versus an external coil (ie, accuracy of lesion detection and localization) is warranted in a larger cohort of patients.

The current study has some limitations. First, this work is a pilot feasibility study with a sample size of 6 volunteers and 20 PCa participants. Of 20 PCa participants, 16 had a biopsy Gleason score of 7, which indicates the presence of intermediate-risk PCa. As a result, our results may not be representative of low (Gleason score ≤ 6) or high-risk disease (Gleason score = 8-10). However, the purpose of this study was to demonstrate the feasibility of using this coil to detect TSC differences between PCa and noncancerous prostate tissue. Second, the sensitivity profile of the butterfly coil is dependent on the separation distance between the 2 external loops. In other words, signal dropoff will be more apparent in participants with thicker pelves. This effect was addressed by normalizing the in vivo images to a ^23^Na sensitivity map obtained from a uniform sodium phantom approximately matching the thickness of each individual pelvis. Finally, the accuracy of our TSC measurements in PCa lesions is limited to the radiologist delineations based on ^1^H mpMRI images, which can be obscured because of the structural heterogeneity of TZ as well as in the presence of conditions like benign prostatic hyperplasia in older patients. For this reason, future studies will use digital whole-mount histopathology to localize and characterize regions of benign prostatic hyperplasia and PCa, and define Gleason grade as the ground truth for in vivo images (mpMRI, ^23^Na MRI, and PET) of PCa participants scheduled for prostatectomy.^27^^,^^28^ Correlations between Gleason grade, TSC, ADC, and other quantitative imaging parameters such as prostate-specific membrane uptake will be explored to establish whether ^23^Na MRI can improve PCa detection and tumor grade characterization in addition to clinical mpMRI and PET.

In conclusion, this work demonstrated the streamlined workflow of ^23^Na MRI for PCa imaging using an external butterfly coil rather than the conventional endorectal coil. For the first time, an external ^23^Na MRI coil was used to quantify and compare TSC in human PCa and noncancerous prostate tissue. Lesions presented with lower TSC and ADC compared to surrounding noncancerous tissue, suggesting TSC is influenced in part by differences in cell density. Future studies will aim to establish the ability of this coil to accurately detect and characterize PCa Gleason grade defined by histopathology as the ground truth.

## Supplementary Material

umae023_Supplementary_Data

## Data Availability

Data generated or analyzed during the study are available from the corresponding author by request.
