# Supplementary material for: Different sodium concentrations of noncancerous and cancerous prostate tissue seen on MRI using an external coil
Source: Radiol Adv. 2024 Sep 30;1(3):umae023. doi: 10.1093/radadv/umae023 (PMC11578593; doi:10.1093/radadv/umae023)
Supplement: umae023_Supplementary_Data [file umae023_Supplementary_Data.zip › 20240925 Supplemental Material.docx]

# Supplemental Materials

## **Appendix 1: Sequence Resolution Assessment**

The observed resolution of the ^23^Na pulse sequence and radiofrequency hardware was based on full width at half maximum (FWHM) analysis of a saline-filled polycarbonate resolution phantom (Figure S1). The phantom (dimensions = 68 × 74 × 50 mm^3^) consisted of equally spaced, solid cylindrical rods (diameters = 4-9 mm) submerged in the center of a container of 50 mM sodium chloride. The sodium signal intensity profile was measured across each row of rods. The FWHM, defined here as the width at half the peak prominence, was measured across each intensity profile using the Signal Processing Toolbox (Version 11.7) in MATLAB (Version 9.14.0.2, R2023a) (Table S1). The minimum resolvable diameter was 6 mm based on visual inspection and a low standard deviation (FWHM = 6.1 mm ± 0.5).

## **Appendix 2: Sensitivity Correction and TSC Quantification**

All ^23^Na MR images were corrected for signal sensitivity drop-offs and converted into TSC maps (Figure S2). To establish the sensitivity profile of the butterfly coil at different coil separation distances, ^23^Na MRI was performed on 50 mM sodium chloride sensitivity phantoms of varying heights (diameter = 20 cm, height = 18-26 cm). These phantoms were stacked to achieve the desired separation distance to correct for the non-uniform sensitive profiles resulting from different pelvic thicknesses of the participants. For these measurements, all three reference vials within the coil housing were replaced with 50 mM sodium chloride vials.

TSC maps for each participant were calculated as follows:

$$TSC={\frac{I_{\mathrm{prostate}}}{I_{\mathrm{phantom}}}}/U$$

where I_prostate_ and I_phantom_ are the voxel signal intensities of the prostate and sensitivity phantom sodium images, respectively, and U is the slope from the linear fit through the mean signal from the three external reference vials. The sensitivity phantom used in this equation was approximately the same thickness as the prostate and had been separately registered to the prostate sodium image using a rigid transformation on 3D Slicer version 5.4.0 (www.slicer.org). The relative uncertainty in TSC (E_TSC_/TSC) was estimated through error propagation:

$$\frac{E_{\mathrm{TSC}}}{\mathrm{TSC}}=\sqrt{\left( \frac{\delta I_{\mathrm{prostate}}}{I_{\mathrm{prostate}}} \right)^{2}{+\left( \frac{\delta I_{\mathrm{phantom}}}{I_{\mathrm{phantom}}} \right)}^{2}+\left( \frac{\delta U}{U} \right)^{2}}\times100\%$$

where δI_prostate_ and δI_phantom_ are the standard deviations in a background ROI of the prostate and phantom sodium images, respectively, and δU is the standard deviation of the slope from the linear fit through the three external reference vials. Prostate sodium images were masked where the relative TSC uncertainty was less than 25%.

Sodium signal drop-off was most apparent in participants with thicker pelvises. The mean pelvic thickness was 20.3 cm ± 1.4 cm in volunteers and 22.3 cm ± 3.3 in patients, requiring phantoms of height 20, 22, 24, 26, and 28 cm to correct for sodium signal inhomogeneity. Following sensitivity correction, the relative uncertainty in TSC was consistently low (< 25%) throughout the prostate and the external reference vials.

## **Appendix S3: Validation of TSC Measurements**

TSC measurements using our external ^23^Na coil and image processing pipeline were validated using a calibration phantom submerged in a 50 mM container of sodium chloride. The phantom consisted of three 50 mM, three 100 mM, and three 150 mM vials of sodium chloride (Figure S3). Prior to sensitivity correction, the vials exhibited decreased sodium signal with distance from the coil. After sensitivity correction, the signal became more uniform across the phantom resulting in a lower standard deviation between each of the three 50, 100, and 150 mM vials (38.7 ± 5.5 mM, 83.4 ± 3.5 mM, 125 ± 10.1 mM). While the measured TSC values slightly underestimated the actual concentrations, we note that this effect is less apparent at lower levels of sodium such as those observed in the prostate and can be further reduced in future studies by using larger external sodium references.

## Supplementary Figure Captions

**Figure S1:** (A) T_2_-weighted (T_2_W) and (B) sodium (^23^Na) MR images of a (C) resolution phantom (68 × 74 × 50 mm^3^) consisting of solid rods (diameters = 4-9 mm). (D) Negative intensity profiles of rods of differing diameters, corresponding to the line segments drawn on (B). ^23^Na MRI spatial resolution was determined empirically based on a full width at half maximum (FWHM) analysis, where FWHM is defined as the width at half the peak prominence.

**Figure S2:** Pipeline for sodium sensitivity correction and tissue sodium concentration (TSC) quantification. (A) The participant’s image was divided by that of a (B) uniform sodium phantom approximately matching the thickness of the individual’s pelvis to generate a (C) sensitivity-corrected image. (D) The slope from the linear fit through the three external reference vials (50, 75, 100 mM) was used to convert signal intensity [arbitrary units] to TSC [mM]. Error bars represent standard deviation. (E) The TSC map was masked where the (F) relative TSC uncertainty map was less than 25%.

**Figure S3:** Validation of tissue sodium concentration (TSC) measurements using a (A) calibration phantom consisting of nine 50, 100, and 150 mM vials of sodium chloride. Fiducial markers consisting of 150 mM vials of sodium chloride were placed in the anterior and posterior coil housing. (B) Prior to sensitivity correction, vials exhibited decreased sodium signal with distance from the coil. (C) Sensitivity correction improves signal uniformity throughout the phantom.

## Supplementary Tables

**Table S1:** Full width at half maximum (FWHM) measurements of individual rods (diameters = 4-9 mm) in a sodium resolution phantom. SD = standard deviation; N = sample size.

| Rod Diameter (mm) |  | | | | | |
| --- | --- | --- | --- | --- | --- | --- |
|  | 4 | 5 | 6 | 7 | 8 | 9 |
| Measured FWHM (mm) |  | | | | | |
|  | 3.8 | 3.9 | 5.5 | 7.1 | 8.2 | 8.9 |
|  | 12.6 | 5.3 | 5.8 | 7.2 | 8.2 | 9.4 |
|  | 4.4 | 5.0 | 6.6 | 7.3 | 8.9 | 9.7 |
|  | 4.5 | 6.7 | 5.8 | 8.0 | 8.5 | 10.2 |
|  | 2.6 | 5.0 | 6.7 | 7.8 |  |  |
|  | 2.3 | 3.7 | 6.3 |  |  |  |
|  |  | 2.9 |  |  |  |  |
| *Mean* | *5.0* | *4.6* | *6.1* | *7.5* | *8.5* | *9.5* |
| *SD* | *3.8* | *1.2* | *0.5* | *0.4* | *0.3* | *0.5* |
| *N* | *5* | *7* | *6* | *5* | *4* | *4* |

**Table S2:** Primary MRI sequences and parameters used in this study.

| Sequence | T_2_-weighted SPACE | Diffusion-weighted | Sodium |
| --- | --- | --- | --- |
| Total scan duration | 7.37 min | 8.06 min | 18.5 min |
| Positioning | Oblique axial | Oblique axial | Straight axial |
| Resolution | 0.72 × 0.72 mm^2^ | 2.5 × 2.5 mm^2^ | 5.0 × 5.0 mm^2^ |
| Field of view | 230 mm | 220 mm | 360 mm |
| Slice thickness | 1.00 mm | 3.0 mm | 5.0 mm |
| TR | 1700 ms | 5100 ms | 50.0 ms |
| TE | 104 ms | 93 ms | 0.50 ms |
| Flip angle | 120 deg | 90 deg | 60 deg |
| b-values | - | 0, 50, 100, 200, 500, 1000, 1500, 2000 s/mm^2^ | - |

**Table S3:** Tissue sodium concentration (TSC) values [mM] for noncancerous tissues of the peripheral zone (PZ), transition zone (TZ), and tumours in the PZ and TZ. Percent differences in TSC (∆TSC) between lesions and noncancerous tissue are listed for participants with prostate cancer (PCa). SD = standard deviation, N = sample size.

| PCa Participant | Noncancerous PZ | Noncancerous TZ | Number of Tumours | PZ Tumours | TZ Tumours | ∆TSC (%) |
| --- | --- | --- | --- | --- | --- | --- |
| 1 | 83.1 | 77.0 | 1 | 66.4 | - | -22.3 |
| 2 | 69.4 | 67.4 | 3 | 74.5, 61.8 | 60.7 | 7.1, -11.5, -10.5 |
| 3 | 88.3 | 114.3 | 1 | 66.1 | - | -28.7 |
| 4 | 82.5 | 79.9 | 2 | 64.2 | 60.4 | -24.9, -27.8 |
| 5 | 73.8 | 77.4 | 1 | 71.5 | - | -3.2 |
| 6 | 73.5 | 85.4 | 1 | - | 75.5 | -12.2 |
| 7 | 79.2 | 101.2 | 2 | 62.5 | 64.3 | -23.6, -44.6 |
| 8 | 56.1 | 57.4 | 3 | 52.0, 61.2 | 58.7 | -7.5, 8.8, 2.3 |
| 9 | 106.3 | 100.3 | 3 | 73.9, 86.1 | 56.2 | -36.0, -21.0, -56.3 |
| 10 | 80.2 | 94.2 | 1 | 71.5 | - | -11.4 |
| 11 | 90.6 | 102.2 | 1 | 60.3 | - | -40.1 |
| 12 | 93.8 | 81.4 | 1 | 124.0 | - | 27.7 |
| 13 | 76.5 | 89.5 | 1 | 77.2 | - | 0.9 |
| 14 | 62.2 | 72.0 | 1 | 49.9 | - | -22.0 |
| 15 | 70.3 | 63.0 | 2 | 66.8 | 49.6 | -5.2, -23.8 |
| 16 | 93.7 | 81.3 | 3 | 89.9 | 71.7, 98.3 | -4.1, -12.6 |
| 17 | 71.0 | 70.6 | 1 | 55.7 | - | -24.2 |
| 18 | 57.3 | 64.2 | 1 | 61.1 | - | 6.5 |
| 19 | 98.0 | 86.5 | 1 | 80.7 | - | -19.5 |
| 20 | 58.3 | 51.8 | 1 | - | 44.9 | -14.3 |
| *Mean* | *78.2* | *80.9* | *-* | *70.3* | *64.0* | *-14.0* |
| *SD* | *14.1* | *16.3* | *-* | *16.0* | *15.1* | *18.2* |
| *N* | *20* | *20* | *31* | *21* | *10* | *31* |
| Volunteer | **Noncancerous PZ** | **Noncancerous TZ** |  |  |  |  |
| 1 | 54.1 | 56.3 |  |  |  |  |
| 2 | 65.2 | 55.0 |  |  |  |  |
| 3 | 75.8 | 74.8 |  |  |  |  |
| 4 | 67.0 | 60.7 |  |  |  |  |
| 5 | 65.2 | 60.6 |  |  |  |  |
| 6 | 83.2 | 86.8 |  |  |  |  |
| *Mean* | *68.4* | *65.7* |  |  |  |  |
| *SD* | *10.0* | *12.5* |  |  |  |  |
| *N* | *6* | *6* |  |  |  |  |

**Table S4:** Apparent diffusion coefficient (ADC) values [×10^-6^ mm^2^/s] for noncancerous tissues of the peripheral zone (PZ), transition zone (TZ), and tumours in the PZ and TZ. Percent differences in ADC (∆ADC) between lesions and noncancerous tissue are listed for participants with prostate cancer (PCa). SD = standard deviation, N = sample size.

| PCa Participant | Noncancerous PZ | Noncancerous TZ | Number of Tumours | PZ Tumours | TZ Tumours | ∆ADC (%) |
| --- | --- | --- | --- | --- | --- | --- |
| 1 | 994 | 869 | 1 | 779 | - | -24.3 |
| 2 | 1054 | 945 | 3 | 786, 1037 | 735 | -29.1, -1.7, -25.0 |
| 3 | 961 | 991 | 1 | 788 | - | -19.8 |
| 4 | 939 | 815 | 2 | 708 | 548 | -28.0, -39.2 |
| 5 | 728 | 686 | 1 | 576 | - | -23.4 |
| 6 | 853 | 840 | 1 | - | 566 | -39.1 |
| 7 | 874 | 975 | 2 | 1037 | 637 | 17.1, -42.0 |
| 8 | 1001 | 996 | 3 | 739, 943 | 738 | -30.1, -5.9, -29.7 |
| 9 | 927 | 857 | 3 | 670, 686 | 463 | -32.1, -29.9, -59.8 |
| 10 | 879 | 903 | 1 | 586 | - | -40.1 |
| 11 | 1084 | 1088 | 1 | 538 | - | -67.3 |
| 12 | 1377 | 1253 | 1 | 1615 | - | 15.9 |
| 13 | 843 | 812 | 1 | 644 | - | -26.8 |
| 14 | 961 | 969 | 1 | 568 | - | -51.5 |
| 15 | 946 | 829 | 2 | 966 | 695 | 2.0, -17.6 |
| 16 | 1267 | 1099 | 3 | 881 | 813, 859 | -35.9, -29.9, -24.5 |
| 17 | 974 | 874 | 1 | 783 | - | -21.8 |
| 18 | 738 | 870 | 1 | 645 | - | -13.5 |
| 19 | 985 | 810 | 1 | 630 | - | -43.9 |
| 20 | 1006 | 886 | 1 | - | 672 | -27.5 |
| *Mean* | *970* | *918* | *-* | *791* | *673* | *-26.6* |
| *SD* | *152* | *127* | *-* | *242* | *123* | *18.7* |
| *N* | *20* | *20* | *31* | *21* | *10* | *31* |
| Volunteer | **Noncancerous PZ** | **Noncancerous TZ** |  |  |  |  |
| 1 | 892 | 900 |  |  |  |  |
| 2 | 1256 | 1060 |  |  |  |  |
| 3 | 732 | 564 |  |  |  |  |
| 4 | 1063 | 1188 |  |  |  |  |
| 5 | 965 | 784 |  |  |  |  |
| 6 | 1183 | 1198 |  |  |  |  |
| *Mean* | *1015* | *949* |  |  |  |  |
| *SD* | *193* | *249* |  |  |  |  |
| *N* | *6* | *6* |  |  |  |  |
